# Lentiviral vector-based therapy in head and neck cancer (Review)

**DOI:** 10.3892/ol.2013.1652

**Published:** 2013-11-01

**Authors:** DEEPAK UPRETI, ALOK PATHAK, SAM K.P. KUNG

**Affiliations:** 1 Department of Immunology, Faculty of Medicine, University of Manitoba, Winnipeg, MB R3E 0T5, Canada; 2 Department of Surgery, Faculty of Medicine, University of Manitoba, Winnipeg, MB R3E 0T5, Canada

**Keywords:** lentiviral vectors, head and neck cancers, immunotherapy

## Abstract

Head and neck squamous cell carcinoma (HNSCC) is the sixth most common neoplasm worldwide. Despite advances in multimodality treatments involving surgery, radiation and chemotherapy, the five-year survival rate has remained at ~50% for the past 35 years. Therefore, the early detection of recurrent or persistent disease is extremely important. Replication-incompetent HIV-1-based lentiviral vectors have emerged as powerful and safe tools for gene delivery. Commonly, HNSCC is a locoregional disease that presents at or close to the body surface. Thus, HNSCC is amendable to intratumoral injections of gene therapy vectors aimed at correcting defects associated with tumor suppressor genes to induce the direct cytotoxicity of cancer cells or immune modulation to promote antitumor immunity. Current investigations analyzing HNSCC gene mutations and stem cell markers and the cancer immunoediting concept are creating exciting therapeutic opportunities for lentiviral and other gene transfer vectors. The present review reports specific examples of the current applications of lentiviral vectors in HNSCC.

## 1. Introduction

Head and neck squamous cell carcinoma (HNSCC) arises in the upper aerodigestive tract and includes mouth, throat, larynx and neck lymph node cancer. HNSCC is the sixth most common neoplasm worldwide. Despite advances in multimodality treatments that involve surgery, radiation and chemotherapy, the five-year survival rate has remained at ~50% for the past 35 years (1–3). Therefore, the follow-up protocols currently in practice do not appear to diagnose treatment failure and recurrence early enough. There is a requirement for the identification of favorable prognostic biomarkers and the development of improved therapy for the treatment of head and neck cancer patients.

In the last three decades, a substantial association, as well as a synergistic relationship, has been found between alcohol and tobacco use and the development of head and neck cancer (4–6). Although the etiology of alcohol-associated head and neck cancer is not fully understood, genetic changes in tumor suppressor genes are likely to attribute to the transformation from normal to cancer cells in the formation and progression of head and neck cancer (7). p53 is widely accepted as a key tumor suppressor gene due to its important roles in regulating the cell cycle, apoptosis and DNA repair (8). Therefore, mutations in p53 are often associated with predisposition to HNSCC (9). Studies by Agrawal *et al* and Stransky *et al* used next generation sequencing techniques to compare exon sequences of all the determined human genes in normal and tumor cells of the same individual. The two studies revealed that the NOTCH gene underwent mutation in 10–15% of HNSCC tumors, thus, representing the second most commonly mutated gene in HNSCC following p53. In head and neck cancer, insufficient NOTCH1 signaling blocks squamous cell differentiation and contributes to the continuous proliferation of these ‘pro-cancer’ cells (10,11). One of the growth factor receptors that is highly upregulated and responsible for the growth and survival of HNSCC is the epidermal growth factor receptor (EGFR) (12–15). Activation of EGFR with its corresponding ligand leads to the activation of downstream signaling pathways, including RAS/MAP, ERK, PI3K/AKT, Janus kinase and phospholipase protein kinase. The simultaneous activation of these pathways attributes to carcinoma growth and survival (16,17). Monoclonal antibodies against EGFR and EGFR tyrosine kinase inhibitors are two types of treatment options currently in use for HNSCC (18). However, not all patients exhibit similar responses to these treatments. It may be argued that additional growth survival pathways, including VEGF, HER2 and HER3 (19,20), may function in an EGFR-independent manner.

Currently, there are two hypotheses that explain the tumorigenic potential of cancer cells in HNSCC. The stochastic model indicates that all cancer cells acquire equal potential to develop tumors. A cancer cell that initiates tumor formation is selected randomly or by its growth advantage acquired under specific microenvironments (21). By contrast, the cancer stem cell model demonstrates that not all cancer cells are tumorigenic. Only a rare population of cells in tumors shares stem cell traits, including self-renewal and multipotency, which exhibit the potential to form tumors (22). In a number of studies, distinctive markers have been reported in head and neck cancer as cancer stem cell markers. Prince *et al* reported that CD44 represented a marker for cancer stem cells in HNSCC, demonstrating that 5,000 CD44^+^ cells were sufficient to form tumors. By contrast, CD44^−^ cells were unable to form tumors (23). Krishnamurthy *et al* reported another ALDH marker associated with stem cell properties *in vivo*. Over 80% of ALDH^+^ CD44 cells developed into a tumor at a dose 10 times lower than that of ALDH-CD44-Lin cells when implanted in mice (24). Thus, identification of these cell types is advantageous for the early eradication of cancer from primary sites and the development of innovative strategies.

## 2. Immunobiology of head and neck cancer

The immune system is composed of a wide network of cells, tissues and organs that protect the host from bacteria, parasites, fungi, viruses and the growth of tumor cells. A rich immunological environment surrounds the site of the head and neck region. Therefore, few tumor cells are successful in ‘escaping’ from the immunological surveillance covered by effector cells, including T cells, natural killer (NK) cells and B cells (25). The surviving tumor cells gain resistance against the immune response and are capable of establishing tolerance to the immune competent environment. Surviving tumor cells not only interfere with the host immune system but are also able to convert the local microenvironment in their favor to evade the host immune system, including T and NK cells, via several mechanisms that include manipulation of its own immunogenicity and production of immunosuppressive molecules (26–28). T cells are important effector lymphocytes that play a vital role in cell-mediated cytotoxicity and regulation of adaptive immunity (29). A decrease in the absolute cell counts of specific T-cell subsets has been observed in HNSCC patients (30,31). Activation of the T-cell receptor (TCR) of cytotoxic T cells requires specific recognition of the MHC class I/peptide complex. Therefore, HNSCC escapes cytotoxic T cell recognition via downregulation of MHC class I molecules (32,33). The CD3ζ chain is a component of the TCR signaling complex and its expression is essential for T and NK cell activation. Downregulation of the expression of the CD3ζ chain molecule is observed in the negative regulation of TCR signaling (34,35). As Th1 responses are favorable for controlling head and neck cancer (10,36,37), production of the immunosuppressive cytokine, IL-10, suppresses the Th1 response and favors the Th2 response, promoting the growth of head and neck cancer (38). The regulatory T cell is an additional T cell subset that suppresses the immune response of other cells and induces tolerance to self-antigens to avoid autoimmunity (39). In head and neck cancer patients, increases in the number of these regulatory cells have been found to correlate with the observed immune suppression (40–42).

The differentiation and functional activities of diverse T-cell subsets are tightly regulated by the maturation and activation status of dendritic cells (DCs) (43–46). Steady state, immature DCs are tolerogenic, whereas mature DCs have acquired the ability to effectively stimulate specific T-cell subsets, including Th1, Th2 and Th17 (47). Of note, a number of defects in DC differentiation and function have been reported in HNSCC patients and are as follows: i) defective differentiation of mature DCs, where the presence of immature myeloid cells has been found to reduce the expression of MHC class II and costimulatory molecules, resulting in the abnormal differentiation of DCs (26,48); ii) impairment of chemotaxis, whereby the migration ability of monocytes has been found to be defective in HNSCC, which may be due to the tumor-derived factors that suppress the migration of monocytes (49); and iii) defects in DC maturation, where the immunosuppressive effects of soluble factors released by HNSCC have been found to cause the impairment of DC function. A number of tumor-secreted factors have been reported, including IL-10, vascular endothelial growth factor and granulocyte macrophage colony stimulating factor (50). These factors function at various levels, including blocking the differentiation of monocytes, and impairing DC maturation and immune tolerance (51–53). NK cells are an additional subset of lymphocytes that highlight an important innate defense against various harmful stimuli, including viruses and tumors (54,55). Recent studies have demonstrated the importance of NK-DC crosstalk in the regulation of antitumor immune responses (56). Defects in DC function in HNSCC may also contribute to the defects in NK cells and the number of NK cells observed in HNSCC malignancies (57).

## 3. The cancer immunoediting concept

The concept of ‘cancer immunosurveillance’ proposed by Thomas highlighted the important role of the immune system in fighting against cancer (58). However, over the past two decades the ‘cancer immunosurveillance’ hypothesis has been challenged by observations that revealed the controversial role of the immune system in the promotion of tumor formation (29). The concept of ‘cancer immunoediting’ has been proposed recently to address antitumor immunity, tumor dormancy and escape mechanisms of immune recognition (59). The cancer immunoediting model consists of three sequential phases (3E phases), elimination, equilibrium and escape. The elimination phase is described with regard to the immune surveillance hypothesis, where the immune system is dominant compared with cancer cells. The immune system recognizes the altered cellular activity and tumor antigens and ultimately, eliminates cancer cells. During this phase, the host is free from cancer and nascent cancer cells are destroyed by the immune system before they may transform into tumors. The equilibrium phase is the second phase in which the immune system and cancer cells equally fight for survival. Tumor growth is maintained and prolonged tumor dormancy creates an environment where a rare, small fraction of tumor cells acquire novel mutations that allow them to resist immune attack and enter the final phase. The escape phase is the final phase in which tumor cells are no longer recognized by the immune system. The continuous pressure of the immune system against the cancer cells switches the cells from the equilibrium phase to the escape phase. The cancer cells lose their tumor microenvironment favoring their growth and effectively blocking immune recognition. Thus, cells that survive throughout the process emerge as tumors and are visible in clinical tests (59,60).

## 4. Emerging concepts in lentiviral vector use for the treatment of HNSCC

The cancer gene therapy approach presents a means to introduce therapeutic/cytotoxic genetic materials into cancer cells to correct defects by replacing/suppressing the effects of the mutated gene or to destroy the cancer cells without harming the surrounding normal cells. Viral gene delivery vector systems, primarily retroviral and adenoviral vectors, have been widely used in animal studies and clinical trials to date (61). With the advances that have been made in the understanding of gene mutations/functions in cancer biology and application of suitable gene delivery systems, the first successful retroviral gene therapy trial for the treatment of melanoma was reported in 2006 (62). In this study, adoptive transfer of autologous T lymphocytes, engineered to encode a TCR specific for tumor-associated antigens, successfully regressed metastatic melanoma in two out of 15 patients. In addition, the great potential of using a gene therapy approach was highlighted for the treatment of cancer patients. Commonly, HNSCC is a locoregional disease that presents at or close to the body surface. Therefore, it is amendable to intratumoral injection of gene therapy vectors that aim to correct defects associated with tumor suppressor genes, induce the direct cytotoxicity of cancer cells (including the suicidal HSV-Tk gene and oncolytic ONYX-015 adenovirus) or induce immune modulation (including MHC-B7) to promote T-cell recognition of tumor cells (63–66).

Replication-incompetent HIV-1-based lentiviral vectors have emerged as powerful and safe tools for gene delivery due to their ability to transduce dividing and non-dividing cells (67–70). This enables the integration of genes into the host genome to sustain stable transgene expression, pseudotyping with vesicular stomatitis virus glycoprotein (VSV-G) or other modified envelope proteins to efficiently transduce the cells of interest (71). These vectors are relatively less immunogenic when compared with adenoviral vectors. In addition, they exhibit a different preference for the sites of vector integration in the host genome DNA when compared with retroviral vectors (72), thus, rendering them less likely to transform specific transduced cells via insertional mutagenesis of the proto-oncogene/tumor suppressor gene. To date, the application of the lentivirus to the transduction of primary immune cells, including hematopoietic stem cells, NK cells and DCs (71,73,74), has been successful, providing an additional platform for the genetic engineering of host immune cells to augment antitumor activities. At present, there are a limited number of studies that have analyzed the use of lentiviral vectors as a tool for the delivery of genes in HNSCC. However, accumulating experimental and preclinical data has highlighted the proof of principle for the treatment of head and neck cancer using lentiviral vectors. The present review focuses on the multiple applications of lentivirus mediated gene therapy for the treatment of head and neck cancer in various experimental settings. As the concepts of RNA interference, cancer stem cells and cancer immunoediting are growing, an exciting opportunity is emerging for the future application of lentiviral vectors in cancer gene therapy and immunotherapy settings.

Commonly, the primary HNSCC tumor is treated with radiation or surgery. Despite intensive local treatment, tumor recurrence and metastases to distant sites is inevitable. Metastatic tumors may therefore require an alternative approach, including chemotherapy and other targeted agent therapies (75). As the majority of patients succumb to their condition due to these two critical effects, targeting these key steps is likely to be important for prolonging the survival of cancer patients (76). Degradation of the extracellular matrix by matrix metalloproteinases (MMPs) facilitates the migration of cancer cells to the surrounding normal cellular environment (77–80), and is consistent with the invasion and metastasis process. These proteases assist the diffusion of cancer cells into the vascular and lymphatic systems via degradation of the tissue structure (81). Targeting these MMPs may be useful for the successful treatment of cancer patients. Sun *et al* silenced MMP-2 expression in laryngeal squamous cell carcinoma using lentiviral vectors. The authors observed that inhibition of these endopeptidases significantly impedes the invasion and growth of laryngeal squamous cancer cells (82). Cisplatin is commonly offered to patients with advanced stage head and neck cancer. However, there are numerous cases where chemotherapy treatment has failed due to the expression of resistance genes in tumors (83). These gene products expel the drugs from the cytoplasm and shorten the duration of chemical exposure to cancer cells. The development of drug-resistant tumor cells accounts for the failure of chemotherapy in the control of cancer cell metastasis. The ABC transporter family has been identified as an anticancer drug transporter family (84,85) and among the transporters, ABCC2 has been shown to be involved in resistance to anticancer drugs, including cisplatin (86). Xie *et al* demonstrated that siRNA targeting ABCC2 mediated by a lentivirus markedly increased the cellular concentration of cisplatin, thereby increasing the sensitivity of cisplatin against the cancer cells and reducing the growth of cancer cells *in vivo* and *in vitro*(83). Chemokine receptors are likely to be involved in the metastasis of tumors and are unregulated in several types of human tumor, including breast, melanoma, prostate and colon (87–89). A study conducted by Hong *et al* demonstrated that the downregulation of CXCR4 using a lentiviral vector induced the antiproliferative and anti-invasive effects in oral cancer cells. In an *in vivo* model, the authors constructed a lentiviral expression vector targeting CXCR-4 mRNA. Using this model, the expression of CXCR-4 was knocked down in two oral squamous cell lines, KB and KB0SSC-25 B. The results showed that the anti-invasive effect was 29.5 and 38.1% in KB and KB0SSC-25B cell lines*,* respectively, compared with that in control vector-infected cells. In addition, CXCR4 knockdown cells grew significantly slower than the vector-infected cells, confirming the antiproliferative effect (90). It is important to investigate whether these gene-silenced cells are likely to inhibit the migration of tumor cells to distant sites, and to identify the impact on the induction of antitumor immune responses *in vivo*. A study by Chen *et al* reported that ALDH1 functions as a putative marker for cancer stem cells in HNSCC (91). In addition, the authors revealed that BMi-1, initially identified as a proto-oncogene, is responsible for maintaining the self-renewal capacity of HNSCC^−^ALDH1^+^ cells. Using the lentivirus expression vector plVRNAi/sh-BMi-1, BMi-1 expression was knocked down in ALDH^+^ cells. BMi-1-silenced ALDH^+^ cells showed stronger chemotherapeutic effects, as well as increased efficacy for radiotherapy, *in vivo*(92).

The dual opposite functions of immune interactions with cancer are being increasingly reported. According to the 3E phases of the cancer immunoediting model, if cancer has progressed beyond the equilibrium phase, the immune system may provide support to the cancer progression process rather than eliminate the cancerous cells. It is therefore important to understand the interactions of the tumor microenvironment, various immune cell types and cancer cells when designing a strategy to augment antitumor responses. In head and neck cancer, the immune system is often compromised due to the production of various suppressive factors (26,27) or malfunction of key immune cells. Head and neck cancer evades the host immune system via specific mechanisms, including the downregulation of HLA class I molecules (93,94) or CD3ζ in T cells. As the CD3ζ chain is a subunit of the TCR complex, essential for signal transduction (25), T cells of HNSCC patients show compromised cytotoxic ability (75,95), decreased proliferation when stimulated with IL-2 (25) and decreased production of proinflammatory cytokines, including IL-2 and IFN-γ (96). Thus, restoration of a functional immune system may be useful for controlling disease progression and tumor growth in HNSCC patients. The design of therapeutic strategies that eliminate primary cancer cells (elimination phase) and augment the host immune system against cancer cells (equilibrium phase) are likely to reduce the growth of primary tumors and spreading of cancer cells to secondary locations. HIV-1 viral protein (vpr) is an accessory vpr required for importing the HIV-1 vpr-integrating complex into the nucleus of nondividing cells (97). In addition, the vpr protein alone induces cell cycle arrest and apoptosis in a variety of mammalian cells (98,99). Our previous study determined the antitumor potential of HIV-1 vpr in an AT-84 mouse model of oral cancer (100). A single intratumoral injection of vpr-expressing lentiviral gene therapy vectors reduced the primary tumor volume significantly within 7–14 days (100) and complete regression of the AT-84 tumor was identified in >40% of animals. In addition, the latter were protected from a secondary challenge of AT-84 tumor cells (100). It is possible that expression of the vpr protein induced apoptosis in the tumor cells and augmented the target recognition by tumor cells. The latter may trigger augmented adaptive anti-AT-84 responses in primary and secondary tumor challenges.

## 5. Concluding remarks

Current investigations analyzing HNSCC gene mutations and cancer stem cell markers and the cancer immunoediting concept establish exciting therapeutic opportunities for lentiviral and other gene transfer vectors. Genetic manipulation of effector immune cell responses at the elimination and equilibrium phases may be consistent with the regression of primary tumors and the restoration/augmentation of antitumor responses in HNSCC patients. Strategies aimed at the direct elimination of tumor cells and/or augmentation of antitumor responses to T cells, including the augmentation of Th1 and suppression of T-cell regulation functions or DC levels, may be useful for the boosting of the immune system to fight HNSCC cancer. The VSV-G has been widely used for pseudotyping lentiviral vector particles due to its stability, high titer and broad cell tropism in transduction. Development of novel envelope proteins for pseudotyping lentiviral vector particles is likely to be consistent with cell-type specific transductions *in vivo*. A Sindbis virus glycoprotein, capable of binding DC-SIGN protein, was created to specifically deliver genes of interest into DCs following the administration of *in vivo* viral vectors (101). Further modification of the Sindbis virus envelope protein to express either a Fc-binding protein A domain, a biotin-adaptor peptide or arginine-glycine-aspartic acid RGD peptide have been shown to support targeted cell type-specific transduction (102–104). The latter may be utilized to target DC or cancer stem cells *in vivo*. An important feature of lentiviral vectors is the ability of the viral integrase protein to integrate the genes of interest into the host genome, consistent with the efficient and long-term gene expression of transgenes in transduced cells. Although the preferred sites of integration appear to vary between the lentiviral and retroviral vectors, concern that the vector integrations may pose a risk of insertional mutagenesis remains. In applications that do not require long-term stable gene expression, integration-deficient lentiviral vectors (that harbor class I mutations of the HIV-1 integrase) for transient transgene expression may also provide a safer alternative to the current lentiviral-based therapies (105–108).
